# A review of the Western Australian keeled millipede genus *Boreohesperus* (Diplopoda, Polydesmida, Paradoxosomatidae)

**DOI:** 10.3897/zookeys.290.5114

**Published:** 2013-04-16

**Authors:** Catherine A. Car, Mark S. Harvey

**Affiliations:** 1 Department of Terrestrial Zoology, Western Australian Museum, Locked Bag 49, Welshpool DC, Western Australia 6986, Australia; 2 Research Associate, Division of Invertebrate Zoology, American Museum of Natural History, Central Park West at 79th Street New York, NY 10024, USA; 3 Research Associate, Department of Entomology, California Academy of Sciences, 55 Music Concourse Drive, San Francisco, CA 94118 USA; 4 Adjunct Professor, School of Animal Biology, University of Western Australia, 35 Stirling Highway, Crawley, Western Australia 6009, Australia

**Keywords:** Morphology, new species, taxonomy, short-range endemics, biodiversity

## Abstract

A taxonomic review of the endemic Western Australian millipede genus *Boreohesperus* Shear is presented in which six species are recognized: the type species, *Boreohesperus capensis* Shear, 1992, from North-West Cape, one new species, *Boreohesperus dubitalis*, from Barrow Island and four more new species from the Pilbara region, *Boreohesperus curiosus*, *Boreohesperus delicatus*, *Boreohesperus furcosus* and *Boreohesperus undulatus*. All six species have highly localized distributions, consistent with being short-range endemics. The nomenclature of the branches of the male gonopod is revised.

## Introduction

All native Australian keeled or flat-backed millipedes (Polydesmida: Paradoxosomatidae) belong to the subfamily Australiosomatinae, distinguishable from other subfamilies by the presence of a tubercle (adenostyle) arising near the base and on the medial surface of the femur of the first leg of the male. All but one (*Mjoebergodesmus* Verhoeff, 1924) Australian genera conform to this definition (Jeekel 1979) and usually the male first leg is incrassate and bears an obvious, well-developed tubercle.

In Western Australia, the described species of paradoxosomatids fall into five genera: *Antichiropus* Attems, 1911, *Boreohesperus* Shear, 1992, *Helicopodosoma* Verhoeff, 1924, *Hoplatessara* Verhoeff, 1928, and *Stygiochiropus* Humphreys & Shear, 1993 (Attems 1911; Humphreys and Shear 1993; Rowe and Sierwald 2006; Shear 1992; Shear and Humphreys 1996; Verhoeff 1924). A single species of *Hoplatessara* has been recorded from just one locality in Western Australia: the occurrence of this genus in the state remains in doubt and will be discussed in detail in a forthcoming paper on the millipedes of the Great Western Woodlands area of Western Australia (Car and Harvey, unpublished data). Shear (1992) described the genus *Boreohesperus* and its first known species,* B. capensis*, based on specimens from Cape Range in Western Australia, where it was collected from cave entrances. He pointed out that these specimens were not modified for cave life but that they merely “found conditions of the caves congenial” (p 777). The genus was tentatively assigned to the tribe Australiosomatini by Shear (1992) on the basis of the structure of the gonopod. He suggested that the presence of only two acropodite branches in *Boreohesperus* implied a relationship with several eastern and southern Australian genera that have confidently been assigned to the Australiosomatini. At this stage, there is no further evidence suggesting that the placement of *Boreohesperus* in the Australiosomatini should be revised and the genus is, therefore, still considered the only confirmed representative of the tribe in Western Australia, with the other Western Australian genera, except *Hoplatessara*, assigned to the tribe Antichiropodini.

There is no standardized terminology for the description of paradoxosomatid gonopods which may be highly modified across different taxa (Jorgensen and Sierwald 2010) and authors over the years have used a number of different terms for the same structures (Rowe and Sierwald 2006). In the majority of publications, these terms have also implied that the various structures of a gonopod are homologous with those of the podomeres of a walking leg, but there has been no research to confirm this suggestion (Mesibov 2005). Shear (1992) described the gonopod of *Boreohesperus capensis* as having a short femorite leading into two branches: the long slender solenomerite bearing the sperm canal, and the shorter rod-like tibiotarsus. Jeekel (1968) identified the tibiotarsus as a process arising on the posterior surface of the gonopod, separate from the solenomerite. In *Boreohesperus*, the structure Shear labeled the tibiotarsus appears to arise on the antero-lateral surface of the acropodite, but he believed this was due to the coiling of the gonopod.

In this paper, we redescribe the genus and describe a new species of *Boreohesperus* from Barrow Island that extends the limits of the genus. We also report on four additional new paradoxosomatid species from the Pilbara region of Western Australia (Figure 9). In this paper, they are described and have been included in the genus *Boreohesperus* as they share the same basic gonopod structure with *Boreohesperus capensis*, but, in the light of these new discoveries, the labelling of the gonopod branches has become difficult. As for *Boreohesperus capensis*, each of these new species has a gonopod that splits into two main branches from a short femorite. We have labeled one branch of the gonopod, the solenomere (S) which is the branch that carries the sperm canal, and was referred to as the solenomerite by Shear (1992). The other branch is called the non-seminiferous branch (NSB) here, and is measured from the centre of the solenomere base (bs) to the tip of that branch. The NSB appears to be an extension of the femorite and seems to be the equivalent of that branch labeled the tibiotarsus by Shear. In this paper, the femorite (F) is measured from the solenomere base (bs) to the distal edge of the prefemur (PF). Other structures which make up the acropodite are referred to only as processes to avoid confusion. Thus, the processes found at the tip of the solenomere are labeled solenomere tip processes (stp); any process on the main body of the solenomere is called a solenomere process (sp); the pointed process arising on the NSB is labeled the ‘nsbp’, and a separate process arising posteriorly from the base of the gonopod in some species is tagged the ‘pp’. This last process is a tibiotarsus in the sense used by Jeekel (1968) but has not been named as such in this paper.

## Material and methods

All of the specimens examined for this study are preserved in 75% ethanol, and are lodged in the Western Australian Museum, Perth (WAM). The specimens of the new species described from the Pilbara region were collected during a joint Department of Environment and Conservation and Western Australian Museum survey of the region, as outlined by George et al. (2009).

The specimens were examined with Leica MZ6 and MZ16A stereo microscopes and the images were generated with a Leica MZ16A automontage imaging system using Leica Application Suite Version 3.7.0 software. Images of whole specimens and their dorsal views were captured first. The left gonopod from each specimen was then removed and a set of images of the gonopod from four different orientations (posterior, anterior, medial and lateral) was captured. Descriptions were compiled with the software package DELTA (Dallwitz 1999) and the map was generated with ArcMap version 9.3.1 (ESRI Inc.)

## Taxonomy

### Order Polydesmida Pocock, 1887 Suborder Strongylosomatidea Brölemann, 1916 Family Paradoxosomatidae Daday, 1889 Subfamily Australiosomatinae Brölemann, 1916 Tribe Australiosomatini Brölemann, 1916 Genus *Boreohesperus* Shear, 1992

#### Boreohesperus

Shear, 1992: 778

http://species-id.net/wiki/Boreohesperus

##### Type species.

*Boreohesperus capensis* Shear, 1992, by original designation.

##### Diagnosis.

Four other genera of australiosomatines, apart from *Boreohesperus*, possess gonopods that are divided into two main branches, namely *Dicladosoma* Brölemann, 1913, *Dicladosomella* Jeekel, 1982, *Oncocladosoma* Jeekel, 1985 and *Somethus* Chamberlin, 1920. *Boreohesperus* may be distinguished from the other genera by the two main branches of its gonopod arising from a relatively short but distinct femorite (e.g. Fig. 3E). In *Dicladosoma*, the two thick squat gonopod branches arise from the prefemur, while in the other three genera, the gonopod is split into the two main branches much more deeply than in *Boreohesperus*, almost to the acropodite base.

##### Description.

Modified from Shear (1992). Twenty body segments, each smooth and unsculptured, with distinct waist between prozonite and metazonite. Transverse sternal cross-impressions deeper than longitudinal. Paranota, if present, small, poorly developed. Normal pore formula. Legs and antennae with no remarkable features. Gonopod with coxa relatively broad and robust; prefemur sub-globose; femorite length approximately one-quarter to one-third of acropodite length; remainder of gonopod split into two branches, a long, slender, slightly undulating seminiferous branch with curving tip, and a shorter, more upright, pointed branch, often with an additional process near its tip.

#### Boreohesperus capensis

Shear, 1992

http://species-id.net/wiki/Boreohesperus_capensis

Figs 1A , 2 , 9

Boreohesperus capensis Shear, 1992: 779, fig. 1.

##### Type material.

Holotype male: Cave 324, Cape Range, Western Australia, Australia, 22°22'34"S, 113°51'25"E, 27 August 1989, M. East (WAM T23659; original number WAM 91/1408).

**Paratypes:** 1 female, same data as holotype (WAM T23660; original number WAM 91/1409); 2 males, same data as holotype except Cave 203 22°26’14"S, 113°54’39"E, 19 July 1989, W.F. Humphreys (WAM T23661-T23662; original numbers WAM 91/1410-1411). Other paratypes, lodged in the Australian Museum (Sydney), the American Museum of Natural History (New York) and the Zoologische Museum (Amsterdam) were not examined for this study.

##### Other material examined.

**Australia: *Western Australia*:** Cape Range, 21°55'S, 114°04'E, May 1965, A. Saar, 1♂, 1♀ (WAM T44397); Cape Range, top of range, S. of Shot-hole canyon (#3243), 22°02'S, 114°01'E, by hand, 26 July 1967, W.F. Humphreys, 1♂ (WAM T71462); East coast plain, 21°57'S, 114°07'E, by hand, 28 July 1993, W.F. Humphreys, R.D. Brooks, 1♀ (WAM T71463); Cape Range, cave C-111, Breakdown Maze, 21°55’08"S, 114°00’17"E, 5 July 1989, R. Wood, M. East, 1♀ (WAM T71464); Cape Range, cave C-111, Breakdown Maze, 21°55’08"S, 114°00’17"E, 5 July 1989, R. Wood, M. East, 1♀ (WAM T71469); Cape Range area, WAWA Bore 43, cave C-499, 21°56'S, 114°06'E, by hand, 7 July 1993, R.D. Brooks 1 juvenile (WAM T71470); Cape Range area, on damp soil near cave C-499 (WAWA Bore 43 Cave), 21°56'S, 114°06'E, by hand, 19 May 1993, W.F. Humphreys, R.D. Brooks, 1♂, 2♀, 17 juveniles (WAM T71471); N-W Cape Peninsula, cave C-21 (#3346), 22°14'S, 113°58'E, 10 July 1989, A. Humphreys, R. Wood, 1♀ (WAM T71472); N-W Cape Peninsula, cave C-21 (#3348), 22°14'S, 113°58'E, 10 July 1989, A. Humphreys, R. Wood, 1♀ (WAM T71473); Cape Range area, 4 m outside cave, C-118 (#399), 22°09'S, 113°59'E, pitfall traps, 23 July 1989, E.C. Pryor, 1♂ (WAM T71474); Cape Range area, outside cave, C-118 (#320), 22°09'S, 113°59'E, wet pitfall traps, 27 July 1989, E.C. Pryor, 1♂, 1♀ (WAM T71475); Cape Range area, cave C-15, 22°13'S, 113°59'E, 13 August 1992, W.F. Humphreys, R.D. Brooks, R. L’Heureux, 1♀ (WAM T71476); Cape Range area, cave C-222, 21°56'S, 114°06'E, by hand, 10 August 1992, W.F. Humphreys, R.D. Brooks, 1♂, 1♀ (WAM T71477); N-W Cape Peninsula, cave C-232 (#4092), 21°56'S, 114°05'E, 10 July 1989, E. Bowra, 1♂ (WAM T71478); N-W Cape Peninsula, cave C-18 (#3544), 22°05'S, 114°00'E, 26 June 1989, B. Vine, M.S. Harvey, R.D. Brooks, 1♀ (WAM T71479); N-W Cape Peninsula, outside cave C-161 (#3065), 22°13'S, 113°58'E, 2 August 1989, E. Pryor, M. East, 1♀ (WAM T71480); N-W Cave Peninsula, cave C-222 (#3869), 21°56'S, 114°06'E, 3 June 1989, A.J. Humphreys, 1♂ (WAM T71481); N-W Cape Peninsula, cave C-177 (#3988), 22°06'S, 113°58'E, 7 July 1989, M. East, R. Wood 1♀ (WAM T71482); N-W Cape Peninsula, cave C-203 (#2623), 22°26'S, 113°55'E, 19 July 1989, B. Jones, 2♀, 1 juvenile (WAM T71483); N-W Cape Peninsula, near cave C-201 (#425), 22°10'S, 113°58'E, 19 June 1990, D. Brooks, 1♂ (WAM T71484); data lost, presumably Cape Range area, 1♀ (WAM T71485); N-W Cape Peninsula, cave C-162 (#3960), 22°09'S, 114°00'E, 20 June 1989, M.S. Harvey, 1♂ (WAM T71486); N-W Cape Peninsula, cave C-68 (#4238), 22°06'S, 113°59'E, 26 June 1989, R. Wood, 1♂ (WAM T71487); N-W Cape Peninsula, cave C-328 (#3098), 22°01'S, 113°56'E, 28 August 1989, M. East, 1♂ (WAM T71488); N-W Cape Peninsula, cave C-107 (#3267), 22°07'S, 114°00'E, 30 June 1989, B. Vine, E. Bowra, 1 juvenile (WAM T71489); N-W Cape Peninsula, Well #2, Charles Knife Road (#381), 22°06'S, 113°59'E, attracted by ants on track, 3 June 1990, J.M. Waldock, 1♀ (WAM T71490).

##### Diagnosis.

**Figure 1.**
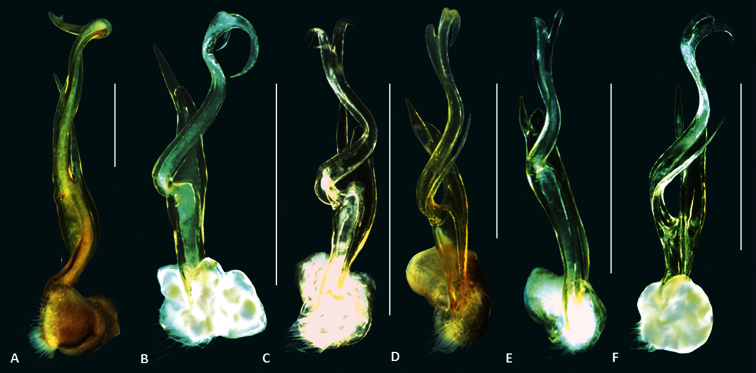
Size comparison of the left gonopod (posterior view), of six *Boreohesperus* species: **A** *Boreohesperus capensis* Shear, 1992 **B** *Boreohesperus curiosus* sp. n. **C** *Boreohesperus delicatus* sp. n. **D** *Boreohesperus dubitalis* sp. n. **E** *Boreohesperus furcosus* sp. n. **F** *Boreohesperus undulatus* sp. n. Scale bars = 0.5 mm.

**Figure 2.**
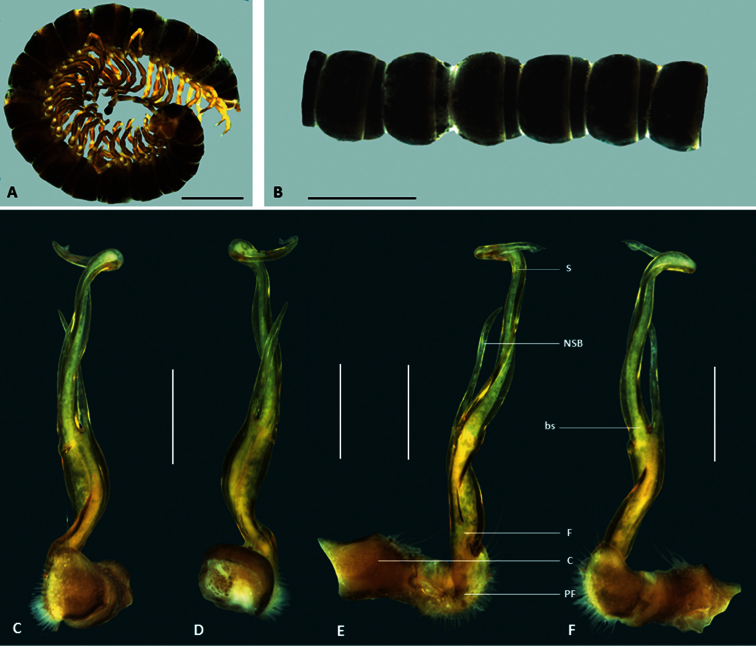
*Boreohesperus capensis* Shear, 1992. **A–B** Male (WAM T44397) habitus: **A** lateral view **B** dorsal view **C–F** Male (WAM T44397) left gonopod: **C** posterior view **D** anterior view **E** medial view **F** lateral view. **bs** solenomere base **C** coxa **F** femur **NSB** non-seminiferous branch **PF** prefemur **S** solenomere. Scale bars: **A–B** = 2 mm; **C–F** = 0.5 mm.

*Boreohesperus capensis* is considerably larger than other species of the genus, measuring approximately 20 mm in length, and 2 mm in mid-body width (Figs 2A–B). Additionally, the solenomere of the gonopod is twisted and undivided at its tip, unlike those of other species, and the non-seminiferous branch of the gonopod in *Boreohesperus capensis* lacks the extra process seen in all other species (Figs 1A, 2C–F).

##### Description.

*Holotype male*: See Shear (1992).

##### Distribution.

This species is restricted to the Cape Range region of Western Australia (Fig. 9).

#### Boreohesperus curiosus sp. n.

urn:lsid:zoobank.org:act:E0E61D2E-4BE0-463B-91AB-E9F3DB5219A3

http://species-id.net/wiki/Boreohesperus_curiosus

Figs 1B , 3 , 9

##### Type material.

Holotype male: 14.5 km NNW. of Mt Elvire, Pilbara Biological Survey site OYE07, Western Australia, Australia, 21°42'39"S, 116°45'57"E, ethylene glycol pitfall traps, 2 October 2005–21 May 2006, CALM Pilbara Survey (WAM T124633).

**Paratype:** 1 female, same data as holotype (WAM T126116).

**Etymology.** This species is named for the shape of the gonopod that is markedly different from those of the other species (*curiosus*, Latin, adjective, odd, different).

##### Diagnosis.

**Figure 3.**
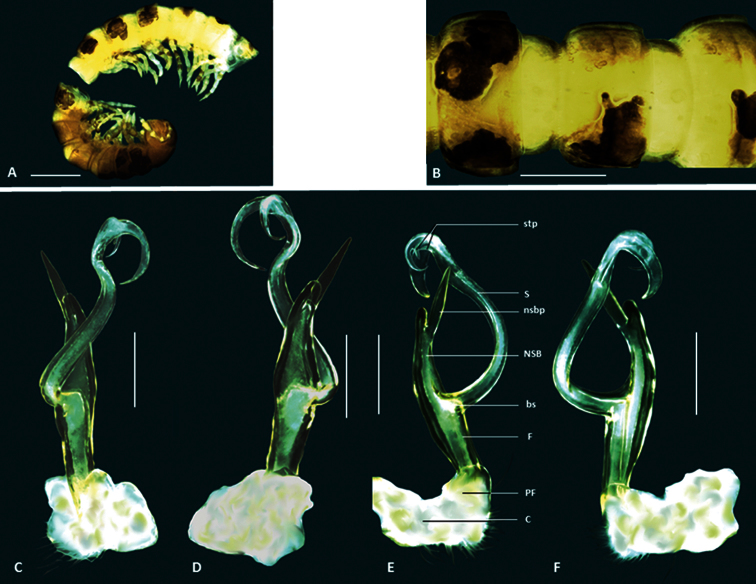
*Boreohesperus curiosus* sp. n. **A–B** Holotype male (WAM T124633) habitus: **A**,lateral view **B** dorsal view **C–F** Holotype male left gonopod: **C** posterior view **D** anterior view **E** medial view **F** lateral view. **bs** solenomere base **C** coxa **F** femur **NSB** non-seminiferous branch **nsbp** non-seminiferous branch process **PF** prefemur **S** solenomere **stp** solenomere tip process. Scale bars: **A** =1 mm; **B** = 0.5 mm; **C–F** = 0.2 mm.

*Boreohesperus curiosus* sp. n. has an easily recognizable gonopod in which the solenomere, when seen in medial or lateral view, curves in an arc (Figs 3E, F) and ends in two large claw-like processes (Fig. 3D). This species also bears two, small, spine-like processes situated at the tip of the solenomere (Fig. 3E).

##### Description.

*Holotype male*: Body approximately 7 mm long; mid-body ring approximately 1 mm wide dorsally, with distinct waist between prozonite and metazonite; legs of moderate length, approximately equal to length of 1 to 2 mid-body rings. Colour bleached by alcohol. Paranota on all but first few body rings small. Sternites, other than those of fifth body ring, with no noticeable features. Anterior spiracles at mid-body flat circular. Antennae distinctly clavate, of moderate length, extending approximately to first body ring behind collum (to body segment 2), antennomeres relatively robust (Figs 3A, B). Gonopod long, extending at least to fifth body ring; coxa (C) much broader than acropodite and approximately 2× as long as broad; prefemur (PF) short, sub-globose; femorite (F) short, one-quarter to one-third length of acropodite, slightly narrower at base, then broadening; non-seminiferous branch (NSB) broadest at solenomere base, then narrowing to form blunt finger shape; process on medial surface of NSB (nsbp) sharply pointed, arising close to NSB tip, and slightly shorter (approximately two-thirds length) than NSB, extending well beyond rounded branch tip; solenomere (S) relatively long and slender, arising midway between NSB tip and prefemur, basal third curving away from NSB and tip curving back towards gonopod midline to form definite arc; solenomere tip divided into two, main pointed claw like forks, with two small spine-like processes (stp) occurring at base of shorter fork when viewed medially; solenomere process (sp) absent; separate posterior process (pp) absent (Figs 1B, 3C–F).

*Female*: similar to male, except for genitalic features.

##### Distribution.

This species in known only from two specimens found at Mt Elvire in the Pilbara region (Fig. 9).

#### Boreohesperus delicatus sp. n.

urn:lsid:zoobank.org:act:69CC2421-755B-4EA1-B1C1-363DEB55E796

http://species-id.net/wiki/Boreohesperus_delicatus

Figs 1C , 4 , 9

##### Type material.

Holotype male: 6 km SE. of Marda Pool, Pilbara Biological Survey site DRW10, Western Australia, Australia, 21°04'11.8"S, 116°12'15.5"E, May 2004, CALM Pilbara Survey (WAM T76070).

**Paratypes:** 1 male, 1 female and 1 immature male, same data as holotype (WAM T126117); 1 male (gonopod only), 11 km ESE. of Marda Pool, Pilbara Biological Survey site DRW07, Western Australia, Australia, 21°03'20.4"S, 116°15'06"E, May 2004, CALM Pilbara Survey (WAM T76065).

##### Etymology.

This species is named for its tiny size and delicate gonopods (*delicatus*, Latin, adjective, dainty).

##### Diagnosis.

**Figure 4.**
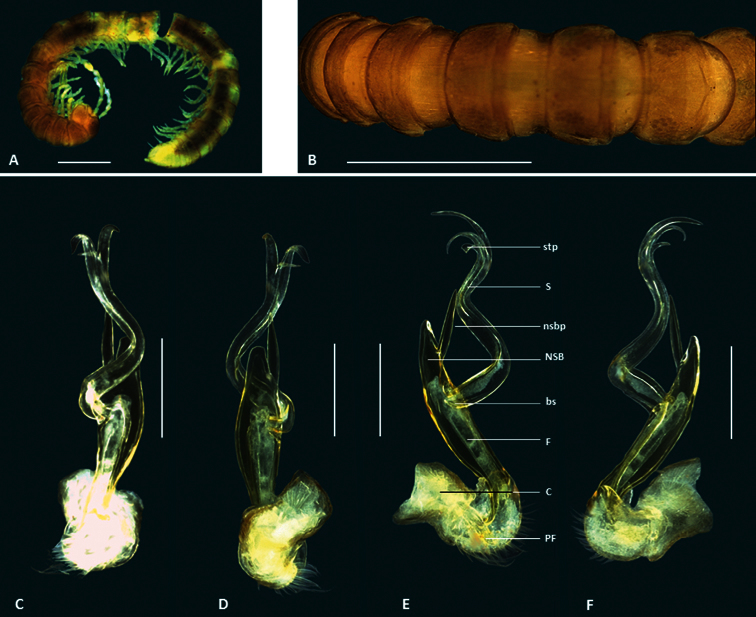
*Boreohesperus delicatus* sp. n. **A–B** Holotype male (WAM T76070) habitus: **A** lateral view **B** dorsal view **C–F** Holotype male left gonopod: **C** posterior view **D** anterior view **E** medial view **F** lateral view. **bs** solenomere base **C** coxa **F** femur **NSB** non-seminiferous branch **nsbp** non-seminiferous branch process **PF** prefemur **S** solenomere **stp** solenomere tip process. Scale bars: **A** = 1 mm; **B** = 1 mm; **C–F** = 0.2 mm.

This species is most similar to *Boreohesperus undulatus* sp. n. but the gonopods of the two species differ in the following ways: (1) in *Boreohesperus delicatus* sp. n. the femorite and non-seminiferous branch (NSB) together form a relatively narrow boat shape (Figs 4E, F) whereas in *Boreohesperus undulatus* sp. n. they form a much broader spindle-shaped branch when viewed medially or laterally (Figs 8E, F); (2) the base of the solenomere of *Boreohesperus delicatus* sp. n. arises approximately midway between the tip of the NSB and the distal end of the prefemur (Figs 4C–F), whereas that of *Boreohesperus undulatus* sp. n. arises closer to the prefemur (Figs 8C, F); and (3) *Boreohesperus delicatus* sp. n. does not possess a posterior process (Fig. 4C), as in *Boreohesperus undulatus* sp. n. (Fig. 8C).

##### Description.

*Holotype male*: Body approximately 7 mm long; mid-body ring approximately 0.75 mm wide dorsally with distinct waist between prozonite and metazonite; legs of moderate length, approximately equal to length of 1 to 2 mid-body rings. Colour bleached by alcohol. Paranota on all but first few body rings small. Sternites, other than those of fifth body ring, with no noticeable features. Anterior spiracles at mid-body flat circular. Antennae distinctly clavate, of moderate length, extending approximately to first body ring behind collum (to body segment 2), antennomeres relatively robust (Figs 4A, B). Gonopod of medium length, extending to posterior edge of fifth body ring; coxa (C) much broader than acropodite and approximately 2× as long as broad; prefemur (PF) short, sub-globose; femorite (F) short, one-quarter to one-third length of acropodite, noticeably narrower at base, then broadening; non-seminiferous branch (NSB) broadest at solenomere base then narrowing to form roughly triangular shape with broadly rounded tip; process on medial surface of NSB (nsbp) slender, arising approximately midway between NSB tip and base of solenomere (bs), similar in length to NSB, extending well beyond branch tip; solenomere (S) relatively long and slender, arising midway between NSB tip and prefemur, forming a distinct ‘S’ shape when viewed in any orientation; solenomere tip divided into two, main pointed ribbon-like forks, with third small spine-like process (stp) seemingly arising between main forks; solenomere process (sp) absent; separate posterior process (pp) absent (Figs 1C, 4C–F).

*Female*: Similar to male, except for genitalic features.

##### Distribution.

This species is known only from the locality of Marda Pool in the Pilbara Region (Fig. 9) where it was found co-occurring with *Boreohesperus undulatus* sp. n.

#### Boreohesperus dubitalis sp. n.

urn:lsid:zoobank.org:act:0D0F45E5-7CB3-435E-9F83-D45D8B4F9B73

http://species-id.net/wiki/Boreohesperus_dubitalis

Figs 1D , 5 , 6 , 9

##### Type material.

Holotype male: Barrow Island, WSW. of Latitude Point, Western Australia, Australia, 20°46'51"S, 115°26'28"E, mostly limestone and rocks, little soil, 11 August 2002, S. Slack-Smith (WAM T57637).

**Paratypes:** 2 males and 3 females, Barrow Island, 500m E. of Base, Western Australia, Australia, 20°49'02.0"S, 115°23'24.4"E, dry pitfall trap, 26 March 2012, R. Teale (WAM T126126).

##### Other material examined.

**Australia: *Western Australia*: Barrow Island:** current airport, helicopter hangar, site N05b, 20°51'50"S, 115°24'23"E, Winkler sac, 1 May 2007, S. Callan, K. Edward, 1♂, 1♀, 1 juvenile (WAM T56353); old administration building, site N23, 20°49'09"S, 115°23'40"E, Winkler sac, 1 May 2007, S. Callan, K. Edwards, 2♀, 3 juveniles (WAM T56354); 20°48'S, 115°24'E, by hand, 31 March 1971, Burbidge, Butler, 1♂, 1 unidentified remains (WAM T73900); 4.5 km N. of Chevron Texaco Camp (NR B21), 20°47'14"S, 115°26'41"E, 8 March-20 May 2006, BIOTA, 1♀ (WAM T83026); Gorgon project, footprint plot GP5, 20°46'59"S, 115°27'03"E, Winkler sac on high limestone flats, 15 March 2006, S. Callan, R. Graham, 4♂, 1♀, 1 juvenile (WAM T121015); Gorgon project, footprint GP7, 20°47'51"S, 115°26'27"E, Winkler sac on limestone ridge to drainage line, 15 March 2006, S. Callan, R. Graham, 1♂ (WAM T121016); Gorgon project, footprint plot CC2, 20°49'02"S, 115°26'24"E, wet pitfall traps on low limestone flats, 10-15 March 2006, S. Callan, R. Graham, 12♂, 1♀ (WAM T121017); Gorgon project, footprint plot GP9, 20°47'59"S, 115°27'00"E, Winkler sac on low limestone ridge, 15 March 2006, S. Callan, R. Graham, 3♂, 2♀, 10 juveniles, (WAM T121018); Gorgon project, footprint plot GP7, 20°47'51"S, 115°26'27"E, wet pitfall traps on limestone ridge to drainage line, 10–15 March 2006, S. Callan, R. Graham, 2♂ (WAM T121019); Gorgon project, footprint plot GP4, 20°47'03"S, 115°27'33"E, Winkler sac on low limestone flats, 15 March 2006, S. Callan, R. Graham, 1♀, 3 juveniles (WAM T121020); Gorgon project, footprint plot GP5, 20°46'59"S, 115°27'03"E, wet pitfall traps on high limestone flats, 10–15 March 2006, S. Callan, R. Graham, 1♂ (WAM T121021); Gorgon project, site 105, 20°48'08"S, 115°26'48"E, Winkler sac, 17 May 2005, S. Callan et al., 1♂, 1 juvenile (WAM T121022); future construction village, 20°49'00"S, 115°26'16"E, wet pitfall traps 17-22 May 2005, S. Callan et al., 1♂ (WAM T121023); Gorgon project, footprint plot GP9, 20°47'59"S, 115°27'00"E, wet pitfall traps on low limestone ridge, 10–15 March 2006, S. Callan, R. Graham, 2♀ (WAM T121024); Gorgon project, footprint plot CC1, 20°49'01"S, 115°26'15"E, wet pitfall traps on valley flats, 10-15 March 2006, S. Callan, R. Graham, 3♂ (WAM T121025); Gorgon project, footprint plot CC2, 20°49'02"S, 115°26'24"E, Winkler sac on low limestone flats, 15 March 2006, S. Callan, R. Graham, 1♂, 1♀, 15 juveniles (WAM T121026); old rubbish dump, 20°47'51"S, 115°20'55"E, Winkler sac on 17 May 2005, S. Callan et al., 1♂ (WAM T121027); Gorgon project, footprint plot CC2, 20°49'02"S, 115°26'24"E, hand collected on low limestone flats, 15 March 2006, S. Callan, R. Graham, 1♀ (WAM T121028); site 22, 20°47'12"S, 115°27'17"E, hand collected, 17 May 2005, S. Callan et al., 2♀ (WAM T121029); Chevron Texaco camp, 20°49'43"S, 115°26'36"E, hand sorted litter, 7 May 2005, S. Callan et al., 1♂ (WAM T121030); 500m E. of Base, 20°49'02.0"S, 115°23'24.4"E, dry pitfall trap, 26 March 2012, R. Teale, 1♂ (WAM T123094); Quarantine Interception from Barrow Island, WAPET Landing, offices, 20°45'29"S, 115°28'19"E, by hand on path between vegetation and building, 8 January 2013, K. Cullen, 1♂ (WAM T126113).

##### Etymology.

This species is named for the fact that, as the first new species of *Boreohesperus* to be discovered, there was initial difficulty in deciding on a genus in which to place it (*dubitalis*, Latin, adjective, to be doubted).

##### Diagnosis.

This species differs from the four other new species because it is noticeably larger, although it is smaller than *Boreohesperus capensis*. In common with *Boreohesperus furcosus* sp. n. and *Boreohesperus undulatus* sp. n., this species carries a process on the main body of the solenomere of the gonopod, but unlike the other species, this process is long and finger-like, extending almost to the solenomere tip (Fig. 6E).

##### Description.

**Figure 5.**
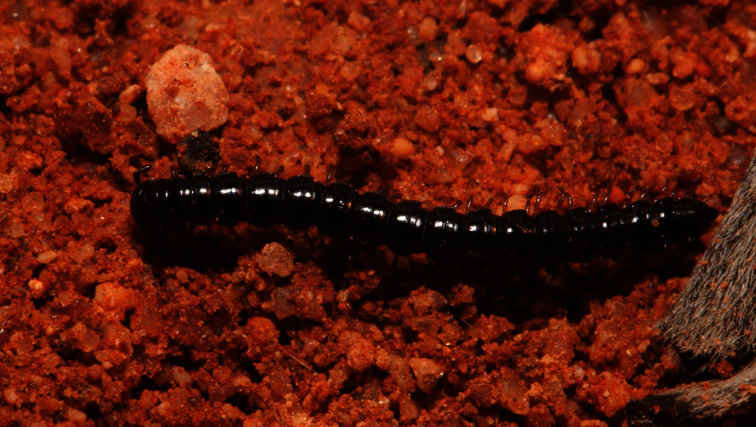
*Boreohesperus dubitalis* sp. n., living male (WAM T126113) (approx. 10 mm long) on Barrow Island. Image courtesy of K. Cullen.

**Figure 6.**
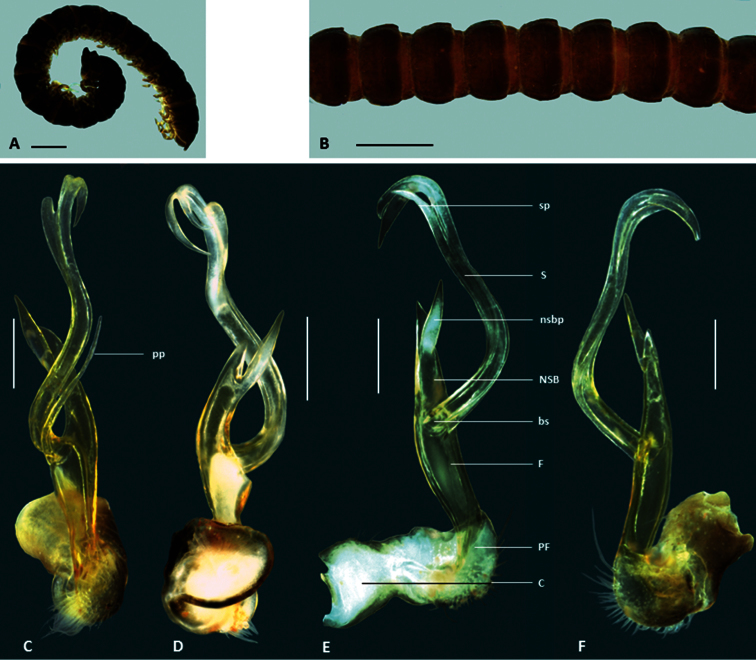
*Boreohesperus dubitalis* sp. n. **A** Male (WAM T123094) habitus, lateral view **B** Holotype male(WAM T57637) habitus, dorsal view **C–F** Holotype male left gonopod: **C** posterior view **D** anterior view **E** medial view **F** lateral view. **bs** solenomere base **C** coxa **F** femur **NSB** non-seminiferous branch **nsbp** non-seminiferous branch process **PF** prefemur **pp** posterior process **S** solenomere **sp** solenomere process. Scale bars: **A** = 1 mm; **B** = 1 mm; **C–F** = 0.2 mm.

*Holotype male*: body approximately 10 mm long; mid-body ring approximately 1.2 mm wide dorsally with distinct waist between prozonite and metazonite; legs of moderate length, approximately equal to the length of 1 to 2 mid-body rings. Colour dark brown overall and legs with coloration similar to that of body. Paranota on all but first few body rings small. Sternites, other than those of the fifth body ring, with no noticeable features. Anterior spiracles at mid-body flat circular. Antennae less obviously clavate, fifth and sixth antennomeres only slightly wider than proximal ones, long, extending beyond body segment 2, antennomeres relatively slender (Figs 5, 6A, B). Gonopod long, extending at least to fifth body ring; coxa (C) much broader than acropodite and approximately 2x as long as broad; prefemur(PF) short, sub-globose; femorite (F) short, one-quarter to one-third length of acropodite, slightly narrower at base, then broadening; non-seminiferous branch(NSB) broadest at solenomere base then narrowing to form pointed finger-like shape; process on medial surface of NSB (nsbp) pointed, arising closer to NSB tip than to solenomere base (bs), and much shorter than NSB; solenomere (S) relatively long and slender, arising midway between NSB tip and prefemur, basal third curving away from NSB and tip curving back towards gonopod midline to form loose arc; solenomere tip divided into two, main pointed ribbon like forks; solenomere process (sp) present, long, finger-like and extending almost to solenomere tip; separate posterior process (pp) arising near solenomere base, long, slender, pointed and approximately half solenomere length (Figs 1D, 6C–F).

*Female*. Similar to male, except for genitalic features.

##### Distribution.

*Boreohesperus dubitalis* sp. n. is endemic to Barrow Island where it is widespread and abundant (Fig. 9). However, due to its restricted distribution of less than 100 km^2^, it clearly represents a short-range endemic species.

#### Boreohesperus furcosus sp. n.

urn:lsid:zoobank.org:act:7D64C1AD-1856-420D-BE41-D32E7074B38E

http://species-id.net/wiki/Boreohesperus_furcosus

Figs 1E , 7 , 9

##### Type material.

Holotype male:9.5 km S. of Mt Minnie, Pilbara Biological Survey site WYW04, Western Australia, Australia, 22°11'19.1"S, 115°33'13.2"E, May 2004, CALM Pilbara Survey (WAM T76078).

**Paratypes:** 4 males and 4 females, same data as holotype (WAM T126118).

##### Etymology.

This species is named for the solenomere of the gonopod that is more branched than those of other species (*furcosus*, Latin, adjective, full of forks).

##### Diagnosis.

**Figure 7.**
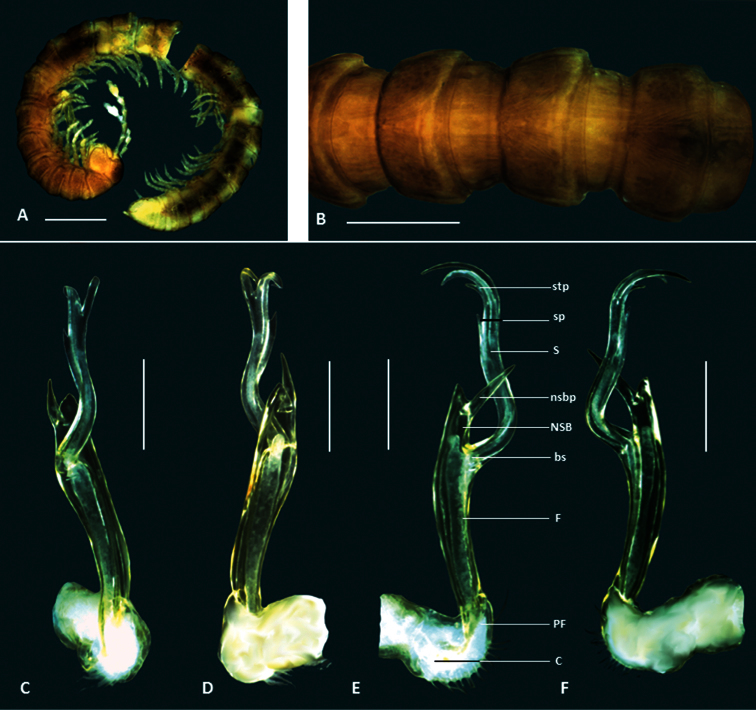
*Boreohesperus furcosus* sp. n. **A–B** Holotype male (WAM T76078) habitus: **A** lateral view **B** dorsal view **C–F** Holotype male left gonopod: **C** posterior view **D** anterior view **E** medial view **F** lateral view. **bs** solenomere base **C** coxa **F** femur **NSB** non-seminiferous branch **nsbp** non-seminiferous branch process **PF** prefemur **S** solenomere **sp** solenomere process **stp** solenomere tip process. Scale bars: **A** = 1 mm; **B** = 0.5 mm; **C–F** = 0.2 mm.

**Figure 8.**
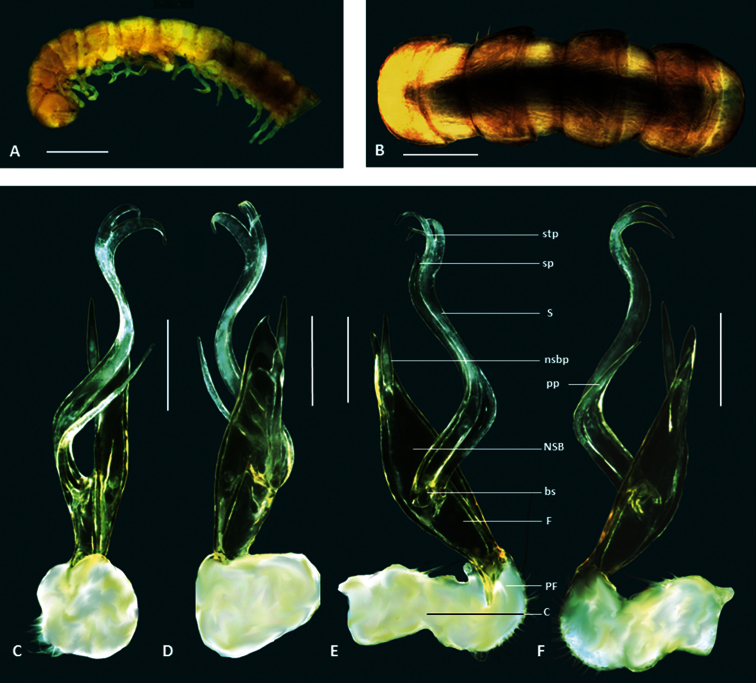
*Boreohesperus undulatus* sp. n. **A–B** Holotype male (WAM T76056) habitus: **A** lateral view **B** dorsal view **C–F** Holotype male left gonopod: **C** posterior view **D** anterior view **E** medial view **F** lateral view. **bs** solenomere base **C** coxa **F** femur **NSB** non-seminiferous branch **nsbp** non-seminiferous branch process **PF** prefemur **pp** posterior process **S** solenomere **sp** solenomere process **stp** solenomere tip process. Scale bars: **A** = 1 mm; **B** = 0.5 mm; **C–F** = 0.2 mm.

This species may be distinguished from the other small species of *Boreohesperus* found in the Pilbara by the more upright solenomere and the presence of two processes on the solenomere of the gonopod which are also found in *Boreohesperus curiosus* sp. n. and *Boreohesperus undulatus* sp. n. In *Boreohesperus curiosus* sp. n., however, these processes arise at the solenomere tip (Fig. 3E) while in *Boreohesperus furcosus* sp. n. and *Boreohesperus undulatus* sp. n., one process is situated near the solenomere tip and the other occurs approximately one third along the solenomere length from the tip (Figs 7E, 8E). Most importantly, *Boreohesperus furcosus* sp. n. lacks the posterior process of *Boreohesperus undulatus* sp. n., and has a noticeably long femorite compared with all the other species.

##### Description.

*Holotype male*:body approximately 8 mm long; mid-body ring approximately 0.75 mm wide dorsally, with distinct waist between prozonite and metazonite; legs of moderate length, approximately equal to the length of 1 to 2 mid-body rings. Colour bleached by alcohol. Paranota on all but first few body rings small. Sternites, other than those of fifth body ring, with no noticeable features. Anterior spiracles at mid-body flat circular. Antennae distinctly clavate, long, extending well beyond body segment 2, antennomeres relatively robust (Figs 7A, B).Gonopod long, extending at least to fifth body ring; coxa (C) much broader than acropodite and approximately 2x as long as broad; prefemur (PF) short, sub-globose; femorite approximately half acropodite length, slightly narrower at base, then broadening; non-seminiferous branch (NSB) short, broad with pointed tip; process on medial surface of NSB (nsbp) sharply pointed, arising close to NSB tip, longer than NSB, extending well beyond branch tip; solenomere (S) relatively slender and upright, arising closer to NSB tip than to prefemur;. solenomere tip divided into two, main pointed ribbon like forks, with third small spine like process (stp) arising at base of main forks; solenomere process (sp) present, short; separate posterior process (pp) absent (Figs 1E, 7C-F).

*Female*: Similar to male, except for genitalic features.

##### Distribution.

**Figure 9.**
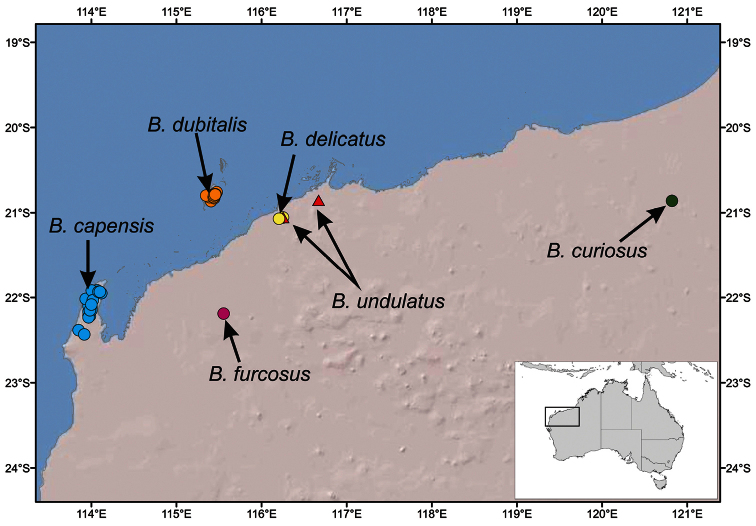
Map of the Pilbara region of north-western Australia showing distributions of *Boreohesperus* species.

This species had been found from only one locality, Mt Minnie, in the Pilbara region of Western Australia (Figure 9).

#### Boreohesperus undulatus sp. n.

urn:lsid:zoobank.org:act:90586ED4-0AAD-4173-A958-D16655A5B3CA

http://species-id.net/wiki/Boreohesperus_undulatus

Figs 1F , 8 , 9

##### Type material.

Holotype male: 3.5 km N. of Karratha Station, Pilbara Biological Survey site DRW05, Western Australia, Australia, 20°51'14.1"S, 116°40'07.9"E, May 2004, CALM Pilbara Survey (WAM T76056).

**Paratypes:** 4 males, 1 female and 1 juvenile, same data as holotype (WAM T126119); 1 male and 1 female, 11 km ESE. of Marda Pool, Pilbara Biological Survey site DRW07, Western Australia, Australia, 21°03'20.4"S, 116°15'06"E, May 2004, CALM Pilbara Survey (WAM T76072).

##### Etymology.

This species is named for the shape of the gonopods (*undulatus*, Latin, adjective, wavy).

##### Diagnosis.

This species is similar to *Boreohesperus delicatus* sp. n., but the gonopod is slightly larger and the shape of the non- seminiferous branch and femorite together is much broader (spindle shaped) than that of *Boreohesperus delicatus* (Fig. 8F). In addition, the gonopod of *Boreohesperus undulatus* sp. n.carries a posterior process (Fig. 8C),lacking in *Boreohesperus delicatus* sp. n. The presence of a posterior process also distinguishes this species from *Boreohesperus furcosus* sp. n., although *Boreohesperus dubitalis* sp. n. also carries this posterior process. *Boreohesperus undulatus* sp. n. is, however, much smaller than *Boreohesperus dubitalis* sp. n., and the solenomere process found on the male gonopod of *Boreohesperus undulatus* sp. n., while present (Fig. 8E), as it is in *Boreohesperus dubitalis* sp. n., is much shorter than the relatively long finger-like solenomere process found on *Boreohesperus dubitalis* sp. n. (Fig. 6E).

##### Description.

*Holotype male*: body approximately 7 mm long; mid-body ring approximately 0.75 mm wide dorsally with distinct waist between prozonite and metazonite; legs of moderate length, approximately equal to length of 1 to 2 mid-body rings. Colour bleached by alcohol. Paranota on all but first few body rings small. Sternites, other than those of fifth body ring, with no noticeable features. Anterior spiracles at mid-body flat circular. Antennae distinctly clavate, of moderate length, extending approximately to first body ring behind collum (to body segment 2), antennomeres relatively robust. Gonopod long, extending at least to fifth body ring; coxa (C*)* much broader than acropodite, and approximately 2× as long as broad; prefemur (PF) short, sub-globose; femorite (F) short, one-quarter to one-third length of acropodite; noticeably narrower at base, then broadening; non-seminiferous branch (NSB) noticeably broadest at solenomere base then narrowing to form broad spindle shape with pointed tip; process on medial surface of NSB (nsbp) pointed, arising closer to NSB tip than to solenomere base (sb), and much shorter than NSB, extending just beyond branch tip; solenomere (S) relatively long and slender, arising closer to prefemur than to NSB tip, forming distinct ‘S’ shape when viewed in any orientation; solenomere tip divided into two, main pointed ribbon like forks, with third small spine-like process (stp) appearing to arise between main forks solenomere process (sp) present, short; separate posterior process (pp) arising near bs, long, slender, pointed and approximately half solenomere length.

*Female*: Similar to male, except for genitalic features.

##### Distribution.

This species has been collected from two localities in the Pilbara: Karratha Station and Marda Pool, situated ca. 50 km apart (Fig. 9).

## Discussion

With few exceptions, paradoxosomatid millipede species in Australia are considered to be short-range endemics, defined by Harvey (2002) as species with natural distributions of less than 10,000 square kilometres. Most maintain very small ranges, well below Harvey’s threshold, for example, species of the genus *Dicladosomella* in south-western New South Wales (Car 2012) and nearly all species of the genus *Antichiropus* in Western Australia (Attems 1911). The genus *Boreohesperus* is confined to Western Australia and, at present, has been found only in a relatively small area of the semi-arid region of that state between latitudes 20°30'S and 22°30'S (Fig. 9). Extensive terrestrial invertebrate surveys have been carried out in Cape Range and the Pilbara region as well as on Barrow Island, and it appears that each paradoxosomatid species, including those of the genus *Boreohesperus*, has a very limited range, with *Boreohesperus dubitalis* sp. n. being endemic to Barrow Island. *Boreohesperus delicatus* sp. n. and *Boreohesperus undulatus* sp. n. are sympatric, both occurring at Marda Pool in the Pilbara and *Boreohesperus curiosus* sp. n. and *Boreohesperus furcosus* sp. n. have each been found at single localities. The rarity of specimens in the collection of the Western Australian Museum, apart from the two species from the intensively sampled Barrow Island and Cape Range caves, suggests that *Boreohesperus* species are rarely active on the surface, and may only emerge from the soil after heavy rains.

## Supplementary Material

XML Treatment for Boreohesperus

XML Treatment for Boreohesperus capensis

XML Treatment for Boreohesperus curiosus

XML Treatment for Boreohesperus delicatus

XML Treatment for Boreohesperus dubitalis

XML Treatment for Boreohesperus furcosus

XML Treatment for Boreohesperus undulatus
